# Association Between Physical Activity and Sedentary Behavior With Depressive Symptoms Among US High School Students, 2019

**DOI:** 10.5888/pcd19.220003

**Published:** 2022-11-17

**Authors:** Charles H. Wang, Nicholas Peiper

**Affiliations:** 1 Noble and Greenough School, Dedham, Massachusetts; 2 Peiper Education and Consulting, LLC, Georgetown, Kentucky

## Abstract

**Introduction:**

The prevalence of depression among US adolescents has increased during the past decade. Previous studies found relationships among physical activity, sedentary behavior, and depression, but more recent information is needed to inform research and practice. We used national surveillance data to assess the association of physical activity and sedentary behavior with depressive symptoms among US high school students.

**Methods:**

This study included 13,526 high school students from the 2019 Youth Risk Behavior Survey. The dependent variable was the presence of depressive symptoms in the past year that lasted almost every day for at least 2 weeks in a row and interfered with usual activities. The independent variables were physical activity (overall activity, muscle-strengthening exercises, participation on sports teams) and sedentary behavior (watching television, using a computer or digital device). We used weighted multivariable logistic regression to evaluate the association of physical activity and sedentary behavior variables with depressive symptoms, while controlling for demographic characteristics and other health behaviors.

**Results:**

The prevalence of depressive symptoms was 36.7%. Participating in physical activity 5 or more days in the past 7 days (adjusted odds ratio [aOR], 0.81; 95% CI, 0.68–0.97) and participating on 1 or more sports teams in the past year (aOR, 0.66; 95% CI, 0.55–0.78) were associated with reduced odds of depressive symptoms. Using a computer or digital device for 3 or more hours per school night was associated with higher odds of reporting depressive symptoms (aOR, 1.61; 95% CI, 1.41–1.85).

**Conclusion:**

Inadequate physical activity and excessive sedentary behavior are associated with depressive symptoms among US high school students. Interventions targeting physical activity and sedentary behavior may be a public health strategy to reduce depressive symptoms in this population.

**Summary**

**What is already known on this topic?**

The prevalence of depression among US adolescents has increased. Depression has severe and potentially lasting effects. Meanwhile, sedentary behaviors, such as screen time, limit physical activity and are common among US adolescents.

**What is added by this report?**

We used a large, nationally representative sample of US high school students to demonstrate that being physically active and spending less time on a computer or digital device are significantly associated with reduced odds of reporting depressive symptoms.

**What are the implications for public health practice?**

Because adolescence is a crucial time for establishing positive health habits, intervention strategies designed to promote physical activity and reduce sedentary behaviors among high school students may be warranted to improve the mental health and the general well-being of this population.

## Introduction

Depression is a common mental disorder among adolescents that is characterized by various psychological symptoms such as persistent sadness, lack of enjoyment, and deterioration of happiness (1). These symptoms start to become pronounced during the transition to high school in middle adolescence and can lead to lack of sleep, disturbance in appetite, a sedentary lifestyle, and even suicide. Depressive symptoms among adolescents in high school have been increasing since approximately 2012, with the most dramatic increases starting in 2015 (2,3). While increases in depressive symptoms have been noted across many age groups since 2012, the prevalence of past-year major depressive episodes increased most rapidly among adolescents in high school (aged 14–17 y) (4,5). These recent trends may be indicative of emergent risk factors and mental health problems among adolescents in high school.

Although considerable research has focused on psychological risk factors such as stress and traumatic childhood experiences, the relationships among physical activity, sedentary behaviors, and depression is a growing area of interest (6,7). National surveillance data indicate that the percentage of adolescents in high school who were physically active for at least 60 minutes per day on 5 or more days in the past week decreased from 2011 to 2019 (8). Similarly, sedentary behaviors such as using digital devices for something other than schoolwork, which limits physical activity, also became more common among adolescents in high school during this time (9,10). According to the American Academy of Child and Adolescent Psychiatry, children aged 8 to 12 years spend roughly 4 to 6 hours per day observing a screen, and adolescents in high school often spend up to 9 hours (11). Excessive screen time can lead to inactivity, which then can lead to weight problems, self-image issues, depressive symptoms, and poor health-related quality of life (12,13). More recently, the COVID-19 pandemic led to decreases in opportunities for regular physical activity and increases in depressive symptoms (14).

Although many factors contribute to adolescents’ mental well-being, dietary behavior has been gaining attention as a key factor in the prevention and management of depression (15). In addition, depressive symptoms among adolescents may also be related to factors such as substance use and behaviors that contribute to violence (eg, feeling unsafe at school, fighting, bullying, cyberbullying), although the relationships may be bidirectional (16–21). For example, depression is associated with substance use among males during adolescence and young adulthood (21), and exposure to acute and chronic stressful events, such as bullying by peers and maltreatment, can be associated with depression among adolescents (20).

Many public health studies have demonstrated associations among physical activity, sedentary behaviors, and depressive symptoms among high school students, although more recent investigations are needed, given recent increases in depressive symptoms among adolescents in high school (2–5). A meta-analysis in 2017 showed that evaluations of physical activity and depression among US adolescents were gender-specific, focused on only one aspect of physical activity or sedentary behavior, or employed small sample sizes (22). By analyzing data from a large sample of the most recent (2019) Youth Risk Behavior Survey (YRBS), this study provides updated evidence on the associations among multiple aspects of physical activity, sedentary behavior, and depressive symptoms among adolescents. By using YRBS’s nationally representative sample, which accurately represents the characteristics of high school students in the US, the results of this study can be generalized to all US students in grades 9 through 12 (23). Our primary objective was to describe the association between different measures of physical activity and sedentary behavior with depressive symptoms among US high school students. We hypothesized that higher levels of physical activity and lower levels of sedentary behavior would be associated with lower levels of depressive symptoms after controlling for demographic characteristics. Our secondary hypothesis was that the associations would hold after additionally controlling for dietary behaviors, substance use, and behaviors that contribute to violence.

## Methods

### Data source and study sample

The YRBS is a cross-sectional national survey that has been conducted by the Centers for Disease Control and Prevention (CDC) since 1991 to monitor health behaviors among US high school students. The YRBS follows local procedures for obtaining parental permission, and students voluntarily complete the surveys. All responses to the survey are recorded anonymously on a computer-scannable questionnaire booklet. CDC’s institutional review board approved this protocol for the national YRBS (23). For this study, we focused on the 2019 national data set that consisted of 13,526 high school students in grades 9 through 12. The 2019 sampling frame included all regular public, Catholic, and private school students, in grades 9 through 12, in the 50 States and the District of Columbia. The school response rate was 75.1%, and the student response rate was 80.3%. Multiplying these 2 percentages (75.1% × 80.3%) together yields an overall response rate of 60.3% (23).

### Study measures

The dependent variable of interest was self-reported depressive symptoms (24). The survey item asked if in the past year students ever felt so sad or hopeless almost every day for 2 weeks or more in a row that they stopped doing some usual activities (yes/no). The main independent variables were physical activity and sedentary behavior (25). Physical activity was measured by using 3 dichotomized variables: being physically active for a total of at least 60 minutes per day on 5 or more days in the past 7 days, doing muscle-strengthening exercises on 3 or more days in the past 7 days, and playing on at least 1 sports team during the past 12 months (8). Sedentary behavior was measured by using 2 dichotomized variables: watching 3 or more hours of television on an average school day and using a computer or digital device for 3 or more hours for something that was not schoolwork on an average school day (9). In addition to demographic variables, we included dietary behaviors, substance use, and behaviors contributing to violence as covariates (15,21). Dietary behaviors included dichotomized variables for skipping breakfast on all days in the past 7 days, not eating any fruits in the past 7 days, and not eating any vegetables (eg, green salad, potatoes, carrots, other vegetables) in the past 7 days (8). Dichotomous variables for substance use were cigarette use in the past 30 days, e-cigarette use in the past 30 days, cannabis use in the past 30 days, alcohol use in the past 30 days, lifetime prescription pain medicine misuse, and lifetime illicit drug use (cocaine, inhalants, methamphetamines, heroin, ecstasy, or hallucinogens) (26). Behaviors contributing to violence included dichotomized variables for school safety concerns in the past 30 days, getting into a physical fight in the past 12 months, being bullied at school in the past 12 months, and being cyberbullied in the past 12 months (18,20).

The operationalizations of depressive symptoms, physical activity variables, sedentary behaviors, and other behavioral risk factors were informed by the extant literature and are consistent with standard definitions and reporting practices developed by CDC (8,9,23). Demographic variables included sex (male/female), grades (9–12), and race and ethnicity (American Indian or Alaska Native, Asian American and Native Hawaiian/Other Pacific Islander, Hispanic or Latino, non-Hispanic Black, non-Hispanic White, and non-Hispanic multiracial). Details about the established reliability and validity of the YRBS are available elsewhere (24,25,27).

### Statistical analysis

Our initial analyses examined weighted frequencies for depressive symptoms, physical activity, sedentary behavior, dietary behavior, substance use, behaviors contributing to violence, and demographic characteristics among US high school students reporting their grade (N = 13,526). We then conducted cross-tabulations to examine bivariate differences in the prevalence of depressive symptoms among demographic groups, physical activity variables, sedentary behaviors, dietary behaviors, substance use, and behaviors that contribute to violence. We conducted pairwise comparisons with Bonferroni correction to determine differences in the prevalence of depressive symptoms within demographic groups. To test the primary hypothesis, we used a multivariable logistic regression model to evaluate the relationship between depressive symptoms with physical activity variables and sedentary behaviors while controlling for demographic variables. For the secondary hypothesis, we used the first model and added dietary behaviors, substance use, and behaviors that contribute to violence as covariates. We calculated adjusted odds ratios (aORs) and 95% CIs for demographic characteristics, physical activity variables, sedentary behaviors, dietary behaviors, substance use, and behaviors that contribute to violence. All analyses incorporated the sample weights and poststratification variables to account for the complex sampling methods of the YRBS. A 2-sided *P* value of ≤.05 denoted significance. We used Stata version 15.1 (StataCorp LLC) for all analyses.

## Results

Overall, students were distributed approximately equally across grades and sex (Table 1). Slightly more than half (51.2%) were non-Hispanic White. The physical activity measures ranged from 44.1% for being active on 5 or more days in the past 7 days to 57.4% for being on 1 or more sports teams. Spending 3 or more hours on a computer or digital device for something other than schoolwork on an average school day was the most commonly reported sedentary behavior (46.1%) followed by watching 3 or more hours of television on an average school day (19.7%). The prevalence of depressive symptoms in the past year was 36.7%.

**Table 1:** Characteristics of High School Students, 2019 Youth Risk Behavior Survey

Characteristic	Overall, % (SE) (N = 13,526)
**Grade**	
9	26.7 (0.6)
10	25.5 (0.4)
11	24.3 (0.6)
12	23.6 (0.6)
**Sex**	
Female	49.4 (0.7)
Male	50.6 (0.7)
**Race and ethnicity**	
American Indian or Alaska Native	0.7 (0.08)
Asian American and Native Hawaiian/Other Pacific Islander	5.4 (1.4)
Hispanic	26.0 (2.3)
Non-Hispanic Black	12.2 (1.1)
Non-Hispanic White	51.2 (2.4)
Non-Hispanic multiracial	4.5 (0.4)
**Physical activity**	
Active ≥5 days in past 7 days	44.1 (1.1)
≥1 Sports team during past 12 months	57.4 (1.5)
Muscle strengthening ≥3 days in past 7 days	49.5 (0.9)
**Sedentary behavior on average school day**	
≥3 Hours of television	19.7 (0.7)
≥3 Hours on a computer or digital device	46.1 (0.8)
**Dietary behaviors in past 7 days**	
Skipped breakfast all days	16.6 (0.7)
No fruits	11.8 (0.7)
No vegetables^a^	7.7 (0.4)
**Substance use**	
Cigarettes in past 30 days	5.9 (0.5)
E-cigarettes in past 30 days	32.7 (1.0)
Cannabis in past 30 days	21.7 (0.1)
Alcohol in past 30 days	29.2 (0.1)
Prescription pain medicine in lifetime	14.2 (0.8)
Illicit drugs in lifetime^b^	14.6 (0.9)
**Behaviors contributing to violence**	
School safety concerns in past 30 days	8.7 (0.6)
Physical fight in past 12 months	21.8 (0.8)
Bullied at school in past 12 months	19.5 (0.7)
Cyberbullied in past 12 months	15.7 (0.5)

a Did not eat green salad, potatoes, carrots, and other vegetables 1 or more times during the past 7 days.

b Lifetime use of cocaine, inhalants, heroin, methamphetamines, ecstasy, or hallucinogens.

Depressive symptoms in the past year increased linearly across grades (33.2% in grade 9 to 39.0% in grade 12) (Table 2). In pairwise comparisons with Bonferroni correction, the prevalence of depressive symptoms was significantly higher in grade 10, grade 11, and grade 12 than in grade 9 (*P* < .05 for all). Nearly half (46.7%) of female students reported depressive symptoms in the past year compared with 26.8% of male students. By race and ethnicity, we found the highest prevalence of depressive symptoms among American Indian/Alaska Native (45.5%) and non-Hispanic multiracial students (45.3%). In pairwise comparisons with Bonferroni correction, we found significant differences (*P* < .01 for all) in the prevalence of depressive symptoms among non-Hispanic Black versus non-Hispanic White students, Hispanic versus non-Hispanic White students, non-Hispanic multiracial versus non-Hispanic White students, Hispanic versus non-Hispanic Black students, non-Hispanic multiracial versus non-Hispanic Black students, Asian American and Other Pacific Islander versus Hispanic students, and Asian American and Native Hawaiian and Other Pacific Islander versus non-Hispanic multiracial students. Among students engaging in physical activity on 5 or more days in the past 7 days, 30.1% reported depressive symptoms in the past year, while approximately one-third of students participating in 1 or more sports teams (32.7%) and engaging in muscle strengthening on 3 or more days in the past 7 days (33.4%) reported depressive symptoms. For sedentary behaviors, the prevalence of depressive symptoms was 40.5% among students who watched 3 or more hours of television in the past 7 days and 42.7% among those who spent 3 or more hours using a computer or digital device for something besides schoolwork on an average school day.

**Table 2:** Weighted Prevalence of Depressive Symptoms Among High School Students, 2019 Youth Risk Behavior Survey

Characteristic	% (SE)
Overall	36.7 (0.8)
**Grade**	
9	33.2 (1.2)
10	37.0 (1.5)
11	37.9 (1.2)
12	39.0 (1.4)
**Sex**	
Female	46.7 (1.1)
Male	26.8 (0.8)
**Race and ethnicity**	
American Indian or Alaska Native	45.5 (6.6)
Asian American and Native Hawaiian/Other Pacific Islander	31.9 (2.1)
Hispanic	40.0 (1.0)
Non-Hispanic Black	31.5 (1.4)
Non-Hispanic White	36.0 (1.0)
Non-Hispanic multiracial	45.3 (3.3)
**Physical activity**	
Active ≥5 days in past 7 days	30.1 (1.0)
≥1 Sports team during past 12 months	32.7 (1.0)
Muscle strengthening ≥3 days in past 7 days	33.4 (1.1)
**Sedentary behaviors on average school day**	
≥3 Hours of television	40.5 (1.3)
≥3 Hours on a computer or digital device	42.7 (1.0)
**Dietary behaviors in past 7 days**	
Skipped breakfast all days	48.8 (1.6)
No fruits	37.5 (1.8)
No vegetables^a^	32.6 (2.0)
**Substance use**	
Cigarettes in past 30 days	58.0 (2.7)
E-cigarettes in past 30 days	48.7 (1.0)
Cannabis in past 30 days	50.4 (1.4)
Alcohol in past 30 days	48.7 (1.2)
Prescription pain medicine in lifetime	59.8 (1.4)
Illicit drugs in lifetime^b^	59.8 (1.9)
**Behaviors contributing to violence**	
School safety concerns in past 30 days	60.0 (2.4)
Physical fight in past 12 months	47.2 (1.3)
Bullied at school in past 12 months	61.9 (1.5)
Cyberbullied in past 12 months	65.1 (1.5)

a Did not eat green salad, potatoes, carrots, and other vegetables one or more times during the past 7 days.

b Lifetime use of cocaine, inhalants, heroin, methamphetamines, ecstasy, or hallucinogens.

In the first multivariable logistic regression model (Table 3, Model 1), students in grade 11 were 21% (aOR, 1.21; 95% CI, 1.03–1.43) more likely than students in grade 9 to report depressive symptoms. Male students were 56% (aOR, 0.44; 95% CI, 0.39–0.49) less likely than female students to report depressive symptoms in the past year. Compared with non-Hispanic White students, Asian American and Other Pacific Islander students were less likely to report depressive symptoms in the past year (aOR, 0.76; 95% CI, 0.58–0.99), while non-Hispanic multiracial students were more likely (aOR, 1.51; 95% CI, 1.12–2.02). For physical activity variables, students engaging in physical activity on 5 or more days in the past 7 days (aOR, 0.81; 95% CI, 0.69–0.93) or participating on 1 or more sports teams in the past year (aOR, 0.71; 95% CI, 0.62–0.82) were less likely to report depressive symptoms in the past year. Students reporting 3 or more hours using a computer or digital device for something other than schoolwork on an average school day (aOR, 1.66; 95% CI, 1.44–1.91) were more likely to report depressive symptoms in the past year.

**Table 3:** Multivariate Associations Between Physical Activity and Sedentary Behaviors With Depressive Symptoms Among High School Students, 2019 Youth Risk Behavior Survey

Characteristic	Adjusted odds ratio (95% CI)	
	Model 1^a^	Model 2^a^
**Grade**		
9	1 [Reference]	1 [Reference]
10	1.18 (0.97–1.43)	1.20 (0.99–1.45)
11	1.21 (1.03–1.43)	1.25 (1.08–1.44)
12	1.16 (0.94–1.44)	1.11 (0.90–1.37)
**Sex**		
Female	1 [Reference]	1 [Reference]
Male	0.44 (0.39–0.49)	0.48 (0.41–0.56)
**Race and ethnicity**		
American Indian or Alaska Native	1.82 (0.88–3.78)	1.53 (0.68–3.45)
Asian American and Native Hawaiian/Other Pacific Islander	0.76 (0.58–0.99)	1.15 (0.86–1.56)
Hispanic	1.12 (0.97–1.30)	1.33 (1.14–1.54)
Non-Hispanic Black	0.79 (0.61–1.01)	0.90 (0.66–1.21)
Non-Hispanic White	1 [Reference]	1 [Reference]
Non-Hispanic multiracial	1.51 (1.12–2.02)	1.49 (1.08–2.04)
**Physical activity**		
Active ≥5 days in past 7 days	0.81 (0.69–0.93)	0.81 (0.68–0.97)
≥1 Sports team during past 12 months	0.71 (0.62–0.82)	0.66 (0.55–0.78)
Muscle strengthening ≥3 days in past 7 days	1.04 (0.92–1.19)	0.99 (0.86–1.13)
**Sedentary behaviors on average school day**		
≥3 Hours of television	1.08 (0.92–1.26)	1.06 (0.87–1.30)
≥3 Hours on a computer or digital device	1.66 (1.44–1.91)	1.61 (1.41–1.85)
**Dietary behaviors in past 7 days**		
Skipped breakfast all days	—	1.45 (1.23–1.71)
No fruits	—	1.00 (0.77–1.31)
No vegetables^b^	—	0.80 (0.65–1.00)
**Substance use**		
Cigarettes in past 30 days	—	1.25 (0.90–1.73)
E-cigarettes in past 30 days	—	1.29 (1.07–1.57)
Cannabis in past 30 days	—	1.15 (0.87–1.51)
Alcohol in past 30 days	—	1.23 (1.02–1.49)
Prescription pain medicine in lifetime	—	1.99 (1.65–2.41)
Illicit drugs in lifetime^c^	—	1.67 (1.36–2.04)
**Behaviors contributing to violence**		
School safety concerns in past 30 days	—	1.93 (1.50–2.49)
Physical fight in past 12 months	—	1.40 (1.18–1.66)
Bullied at school in past 12 months	—	2.13 (1.77–2.57)
Cyberbullied in past 12 months	—	2.14 (1.72–2.66)

Abbreviation: — , does not apply.

a Respondent’s demographics were controlled for in Model 1; Model 2 included demographics, dietary behaviors, substance use, and behaviors contributing to violence.

b Did not eat green salad, potatoes, carrots, and other vegetables one or more times during the past 7 days.

c Lifetime use of cocaine, inhalants, heroin, methamphetamines, ecstasy, or hallucinogens.

In the model controlling for dietary behaviors, substance use, and behaviors contributing to violence in addition to demographics (Table 3, Model 2), we found inverse associations between engaging in physical activity on 5 or more days in the past 7 days (aOR, 0.81; 95% CI, 0.68–0.97) and participating on 1 or more sports teams in the past year (aOR, 0.66; 95% CI, 0.55–0.78) with depressive symptoms; these associations were similar to those found in the first model. The increased odds of depressive symptoms among students reporting 3 or more hours of computer or device use for something that was not schoolwork on an average school day (aOR, 1.61; 95% CI, 1.41–1.85) were also comparable to the increased odds found in the first model. E-cigarette use (aOR, 1.29; 95% CI, 1.07–1.57), alcohol use (aOR, 1.23; 95% CI, 1.02–1.49), prescription pain medicine use (aOR, 1.99; 95% CI, 1.65–2.41), and illicit drug use (aOR, 1.67; 95% CI, 1.36–2.04) were associated with increased odds of depressive symptoms. School safety concerns in the past 30 days (aOR, 1.93; 95% CI, 1.50–2.49), getting into a physical fight in the past 12 months (aOR, 1.40; 95% CI, 1.18–1.66), being bullied at school in the past 12 months (aOR, 2.13; 95% CI, 1.77–2.57), and being cyberbullied in the past 12 months (aOR, 2.14; 95% CI, 1.72–2.66) were associated with increased odds of depressive symptoms.

## Discussion

This study provides updated data on the associations between physical activity and sedentary behavior with depressive symptoms among a nationally representative sample of high school students. In support of our main hypothesis, we found that after controlling for respondent demographic characteristics, being active on 5 or more days in the past 7 days and being a member of 1 or more sports teams were associated with reduced odds of reporting depressive symptoms in the past year. Similarly, spending 3 or more hours using a computer or digital device for something other than schoolwork was associated with increased odds of reporting depressive symptoms. The secondary hypothesis was also supported: the associations observed under the first hypothesis remained significant after controlling for demographic characteristics as well as dietary behaviors, substance use, and behaviors contributing to violence.

Consistent with current literature, our study demonstrated that physical activity may be a protective factor for depressive symptoms among adolescents. Physical activity may exert protective effects through psychosocial and behavioral mechanisms. For example, several hypothetical frameworks propose that well-being is achieved by satisfying basic psychological needs for social connectivity, autonomy, self-acceptance, environmental mastery, and purpose in life (15). Physical activity, specifically participating on sports teams, may facilitate opportunities for social connectivity among adolescents through cooperation and shared goals. Being able to follow a team schedule, listen to coaches, and participate in team workouts and competitions facilitates physical changes which can enhance self-perception (self-acceptance), discipline (purpose in life), and independence (autonomy) (15). Consistent physical activity may also reduce screen time and create more interactions with nature (12–15), which can potentially increase overall well-being.

Similar to psychosocial mechanisms, behavioral mechanisms are improved by increasing physical activity. Specifically, physical activity may improve sleep volume and quality and coping and self-regulation skills. Participation in physical activity is highly recommended for adolescents experiencing malaise and fatigue (28). It is reasonable to assume that energy expenditure during the day can improve sleep patterns, which may be associated with improved mental health. Therefore, adolescents who are physically active at least 5 days per week may have a great probability of developing better sleep habits, as their fatigued bodies will need ample rest. Participation in physical activity can lead to development of self-regulation and coping skills. For example, many physical activity programs and sports necessitate a healthy diet. Combining regular physical activity and a self-regulated healthy diet may increase self-efficacy and resiliency, which may in turn reduce the likelihood of developing depressive symptoms (29,30). Moreover, physical activity and related health behaviors may be associated with well-being through beneficial physiological mechanisms such as increases in hormones and growth factors (eg, endorphins, brain-derived neurotrophic factor), immune function, and anti-inflammatory effects (31).

Our findings on sedentary behavior and self-reported depressive symptoms among high school students are consistent with previous literature. For example, a systematic review based on a mix of cross-sectional and longitudinal studies of the association between sedentary behavior and mental health among adolescents provided strong evidence for the positive association between screen time and depressive symptoms among adolescents (32). Excessive sedentary behavior, such as watching 3 or more hours of television on an average school day, can result in bad sleep habits, lower grades, weight and mood problems, and less time with family and friends (6). Along with sedentary behavior, skipping breakfast and other unhealthy dietary behaviors, which sedentary behavior indirectly encourages, may result in malnutrition, consumption of low-nutrient foods such as processed snacks or fast foods, and obesity or weight gain (33). Regular physical activity is correlated with less sedentary behavior and healthier eating choices, potentially avoiding these problems.

Our study has several strengths. It adds to the existing body of literature by analyzing data from a nationally representative sample of high school students in the 2019 YRBS to study the associations between physical activity, sedentary behaviors, and depression among adolescents. Our results, therefore, are generalizable to all students in grades 9 through 12 enrolled in public and private schools in the 50 states and the District of Columbia (23) Our study also provides updated evidence on the associations between physical activity, sedentary behaviors, and depressive symptoms during a period characterized by dramatic increases in depressive symptoms among adolescents in high school (2–5).

Our study also has several limitations. The cross-sectional design of this study precludes causal relationships between the observed associations between physical activity, sedentary behavior, and depressive symptoms (34). Bidirectional associations are possible such that adolescents with higher levels of depressive symptoms may be less likely to engage in physical activity and more likely to engage in sedentary behaviors. Another limitation is the use of existing data from the YRBS, which depends on the use of single-item measures with limited clinical and diagnostic utility. For example, although the question on depressive symptoms has demonstrated reliability and validity, it is not clinically calibrated for detecting major depressive disorder. Other questions about physical activity and sedentary behavior may also be too general to yield more specific inferences about the types of joint activities students may commonly engage in on school days (eg, exercising while watching television or using a computer or digital device). In addition, the YRBS is administered in schools, and thus, does not include adolescents who have dropped out or been expelled, 2 populations more likely than populations that have stayed in school to engage in unhealthy health behaviors. Lastly, the YRBS is based on self-reported data and may be subject to recall and social desirability biases, especially for items asking about behaviors and cognitions beyond 1 month.

Our study provides updated evidence of the association between physical activity and sedentary behavior with depressive symptoms among high school students in the US. On the basis of these findings, we note several implications for policy and program development. Since high school is a crucial time for establishing healthy behaviors, school-based interventions should continue to focus on physical activity and sedentary behavior as ways to improve both physical and mental well-being (35,36). Universal prevention interventions could focus on educating students on the mental health benefits of physical activity and providing resources for students who are interested in team-based physical activity (37). Other interventions could be improved by integrating research about the social contexts that foster problematic sedentary and dietary behaviors, such as engagement with online content that promotes body dysmorphia and harmful gender norms (38,39). In addition, the associations between depressive symptoms and substance use and behaviors contributing to violence warrant continued attention in public health interventions for adolescents (40,41). Promoting physical activities and reducing sedentary behaviors in the daily lives of adolescents may reduce the number of adolescents who experience depressive symptoms and improve the general well-being of adolescents in high school.
